# Methodological aspects of ^99m^Tc-sestamibi guided biopsy in breast cancer

**DOI:** 10.1007/s40336-016-0201-z

**Published:** 2016-07-16

**Authors:** A. Collarino, R. A. Valdés Olmos, A. F. van der Hoeven, L. M. Pereira Arias-Bouda

**Affiliations:** 1 Section of Nuclear Medicine, Department of Radiology, Leiden University Medical Center, Albinusdreef 2, 2333 ZA Leiden, The Netherlands; 2 Interventional Molecular Imaging Laboratory, Department of Radiology, Leiden University Medical Center, Albinusdreef 2, 2333 ZA Leiden, The Netherlands; 3 Department of Nuclear Medicine, The Netherlands Cancer Institute, Antoni van Leeuwenhoek Hospital, Plesmanlaan 121, 1066 CX Amsterdam, The Netherlands; 4 Department of Nuclear Medicine, Alrijne Ziekenhuis, Simon Smitweg 1, 2353 GA Leiderdorp, The Netherlands

**Keywords:** Molecular breast Imaging, Breast-specific γ-imaging, ^99m^Tc-sestamibi, Radioguided-biopsy, Breast cancer

## Abstract

**Purpose:**

This review aims to discuss the methodological aspects of dedicated molecular breast imaging (MBI) using ^99m^Tc-sestamibi as radiotracer to guide biopsy of occult or unclear breast lesions on mammography (MG) and ultrasound (US) that are suspicious on MBI (BI-RADS criteria 4 and 5), including its advantages, limitations and future clinical applications.

**Methods:**

Literature search was performed using the PubMed/MEDLINE database and “^99m^Tc-sestamibi”, “biopsy” and “breast cancer” as keywords. The search was restricted to English language.

**Results:**

There are few studies on ^99m^Tc-sestamibi guided biopsy methods; to our knowledge, no full studies have yet been reported on clinical validation of this new biopsy procedure. This review describes technical aspects of ^99m^Tc-sestamibi guided biopsy and discusses the advantages and limitations of this procedure in comparison with MG, US and MRI-guided biopsy.

**Conclusions:**

MBI-guided biopsy appears to be a complementary modality and is principally indicated in the case of occult or unclear breast lesions on MG/US, that are suspicious on MBI. The future indication is in targeted biopsies in patients with large heterogeneous tumours. Further studies are needed to define the accuracy of this biopsy procedure.

## Introduction

Breast cancer (BC) is the most common cancer type in women with an estimated 246.660 new cases and 40.450 deaths in the United States, in 2016 [1]. Mammography (MG) is the imaging modality of reference in screening and diagnosis of BC [2]. However, MG has an overall sensitivity of 78 %, decreasing to 48–64 % in women with dense breasts [3]. Ultrasonography (US) is the most common adjunct imaging modality, improving the sensitivity to 78 % when used together with MG in women with dense breasts [4]. However, breast US is associated with a higher callback rate and false-positive biopsy rate [5]. Due to the limitations of both modalities, magnetic resonance imaging (MRI) may be used as an adjunct modality. MRI is, for example, recommended as an adjuvant screening modality in high-risk women [6], increasing the detection rate to 9.5 per 1000 women-years at risk [7] with a sensitivity of 71–92 % and a specificity of 79–86 % [8, 9]. However, breast MRI is costly and limited in women with claustrophobia, obese patients and patients with renal failure [10]. In addition, in the clinical setting MRI shows a relatively low specificity and positive predictive value [11] leading to a high rate of unnecessary biopsies. In the last few years, molecular breast imaging (MBI), also called breast-specific γ-imaging (BSGI), has been introduced as an adjunct modality in BC detection. MBI is a functional tool based on the use of ^99m^Tc-sestamibi as tumour tracer [12]. Recently, a ^99m^Tc-sestamibi MBI-guided biopsy system has been developed, applicable in patients with suspicious breast lesions on MBI (BI-RADS criteria 4–5), which are occult or unclear on MG/US [13]. We performed a search of the literature in PubMed/MEDLINE database using “^99m^Tc-sestamibi” AND “biopsy” AND “breast cancer” as keywords. The search was restricted to English language. The references of the retrieved articles were examined to identify additional articles. The aim of this review is to discuss the methodological aspects of this novel radioguided-biopsy method, including its advantages, limitations and future clinical applications.

## ^99m^Tc-sestamibi MBI technique and interpretation

Increased uptake of ^99m^Tc-sestamibi in breast cancer cells is based on increased vascularity and cytoplasmic mitochondrial density and activity [14, 15]. However, overexpression of multidrug resistance membrane proteins (Pgp and MRP1) and anti-apoptotic Bcl-2 protein of the outer mitochondrial membrane can limit retention of ^99m^Tc-sestamibi in tumour cells [16]. In 2002, the first study described the performance of this functional breast-dedicated modality in patients with breast tumours [17]. Since then, MBI has been validated in several studies [18]. In screening studies in women with dense breasts and increased BC risk, the addition of MBI to MG significantly increased sensitivity to 91 % with a detection rate of 11–12 per 1000 screened women [19, 20]. A recent meta-analysis, including 19 studies, showed that MBI has a sensitivity of 95 % and specificity of 80 % in detecting BC. Additionally, the authors reported that MBI detected MG-occult breast lesions in 4 % and additional lesions in 6 % of patients with suspicious MG or proven breast lesions [18]. ^99m^Tc-sestamibi-MBI refers to functional imaging of the breast using a breast-dedicated high-resolution, small field of view (FOV) gamma camera; the images, based on the detection of increased uptake of ^99m^Tc-sestamibi in the tumour in comparison to normal tissue, are independent of breast density. The original MBI system still employs a single detector with a 20 × 15 cm FOV, containing an array of sodium iodide (NaI) crystals (3 × 3 mm pixel size) coupled to position sensitive photomultiplier tubes (PSPMTs). Most literature reports have been based on the use of a single-head system (Dilon 6800^®^, Dilon Technologies, Newport News, VA). In recent years, dual-head detection became available following the introduction of the MBI devices Discovery NM750b (GE Healthcare, Milwaukee, WI) and LumaGem 3200 s (Gamma Medica, Inc., Northridge, CA) which employ two opposite cadmium-zinc-telluride (CZT) detectors with small FOV (24 × 16 resp. 20 × 16 cm) and 2.5 resp. 1.6 mm pixel size; these devices are aimed to provide better energy resolution [21]. A summary of the commercially available MBI devices is shown in Table 1.

**Table 1:** Summary of characteristics of commercially available devices for molecular breast imaging

Camera	Single/dual-head	Detector	FOV (cm)	Pixel size (mm)	Light detection	Biopsy-guidance	Additional information
Dilon 6800 (Dilon Diagnostics)	Single-head	NaI	20 × 15	3 × 3	PSPMTs	FDA-approved	http://www.dilon.com
Dilon 6800 Acella (Dilon Diagnostics)	Single-head	CsI	25 × 20	3.2 × 3.2	PSPMTs	FDA-approved	http://www.dilon.com
GE Discovery NM750b MBI (GE Healthcare)	Single/dual-head	CZT	24 × 16	2.5 × 2.5	Semiconductor	In development	http://www3.gehealthcare.com
LumaGEM 3200s (Gamma Medica)	Dual-head	CZT	20 × 16	1.6 × 1.6	Semiconductor	N/A	http://www.gammamedica.com

*FOV* field of view, *Nal* sodium iodine, *PSPMTs* position sensitive photomultiplier tubes, *FDA* the food and drug administration, *Csl* cesium iodine, *N/A* information not available, *CZT* cadmium zinc telluride

**Fig. 1:**
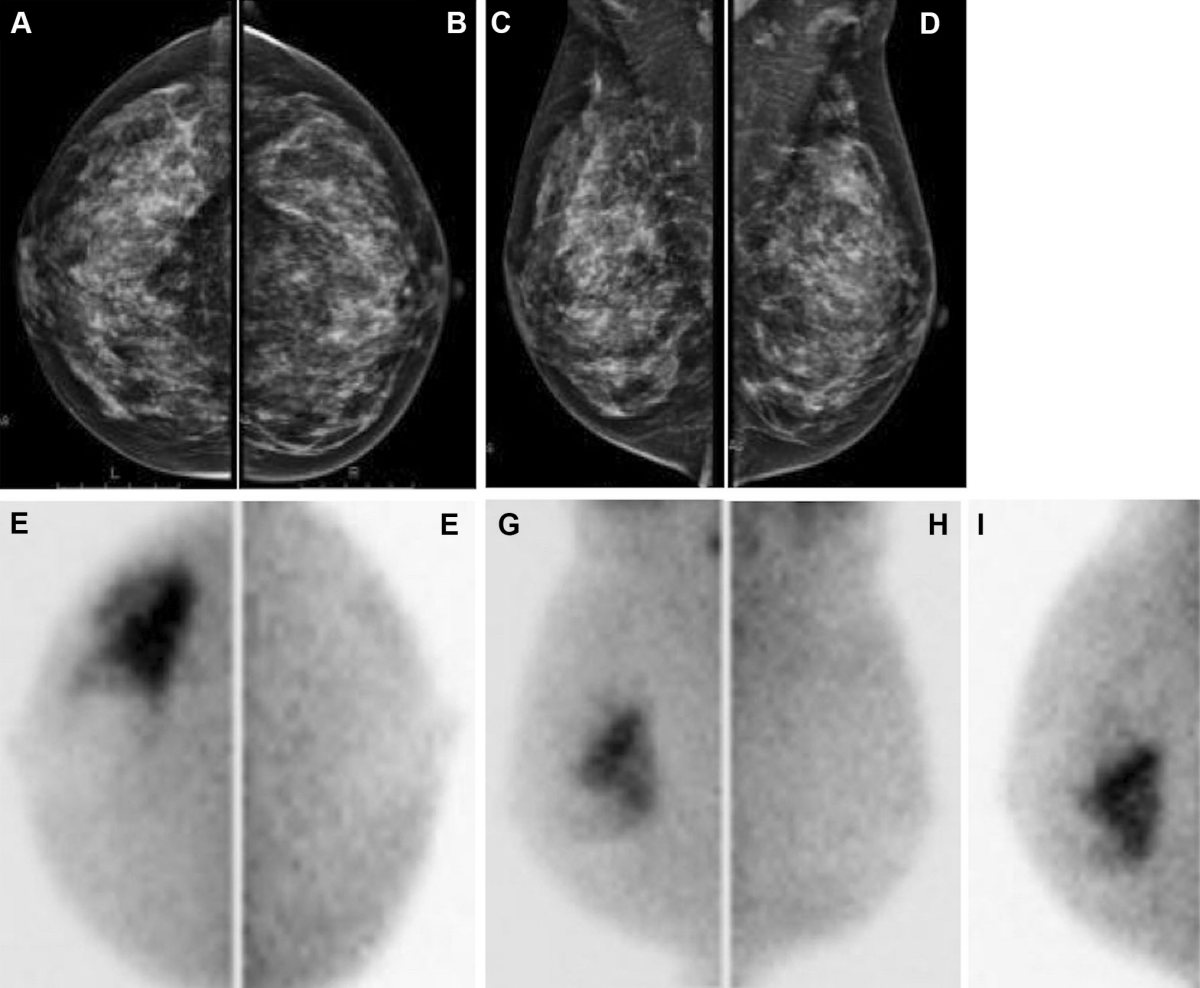
Mammography and MBI in a 47-year old female with dense breasts. Mammography showed no abnormalities (BI-RADS I) in *right* (**a**) and *left* (**b**) craniocaudal views and in *right* (**c**) and *left* (**d**) mediolateral oblique images. MBI showed suspicious uptake (BI-RADS V) in *right* craniocaudal (**e**), lateral-oblique (**g**) and additional lateral views (**i**). Histopathological examination revealed invasive adenocarcinoma

**Fig. 2:**
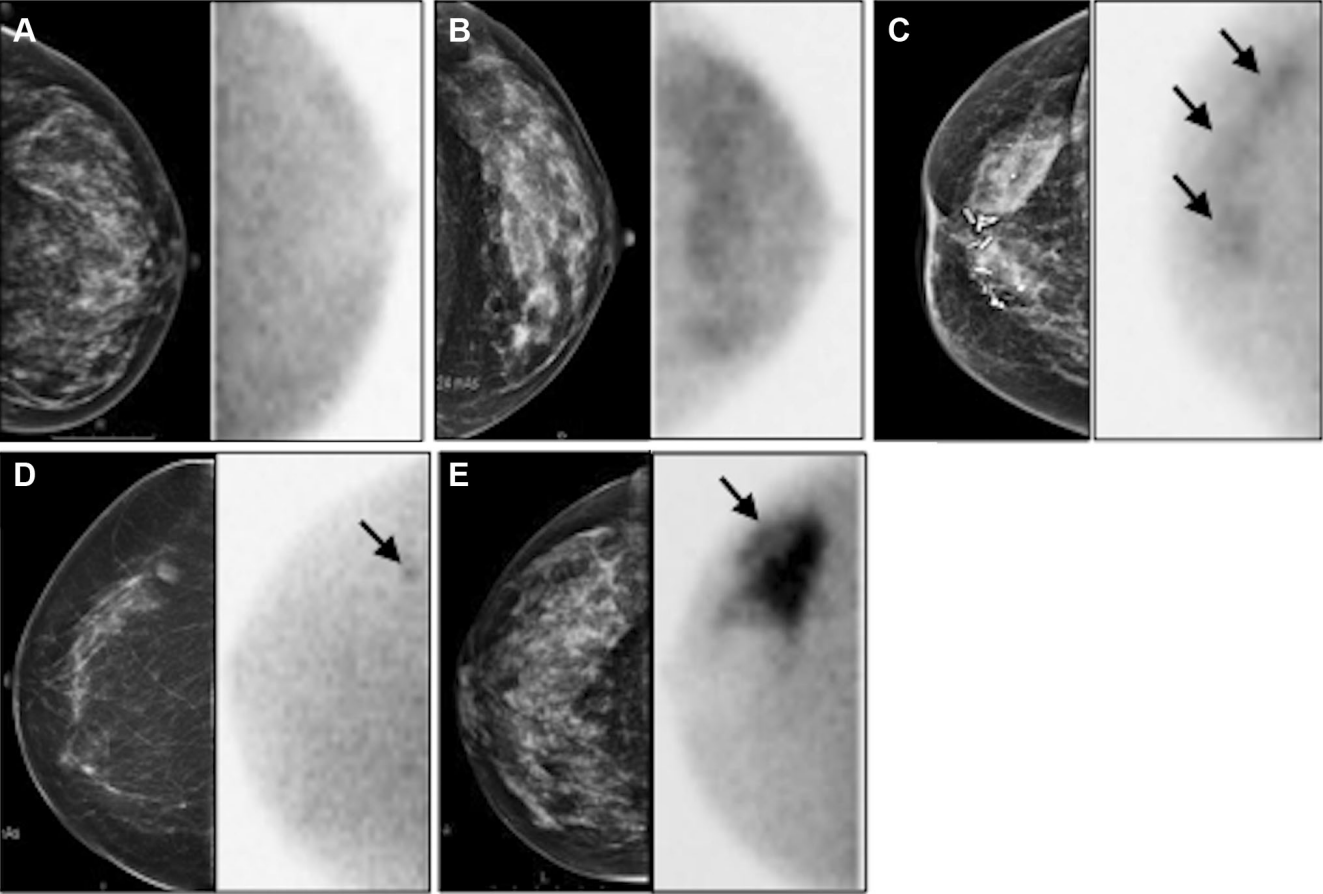
Examples of MBI according to BI-RADS classification [12] displayed together with corresponding mammography.* Left* craniocaudal view (**a**) showing homogeneous uptake (BI-RADS I); *left* craniocaudal view (**b**) showing diffusely increased uptake (BI-RADS II); *right* craniocaudal view (**c**) showing multiple patchy areas of uptake (BI-RADS III) pointed by *arrows*; right craniocaudal view (**d**) showing small focal area of increased uptake (BI-RADS IV, *arrow*); *right* craniocaudal (**e**) showing intense uptake (BI-RADS V, *arrow*)

In both single-head and dual-head MBI devices, the patient is seated during the entire study and the breast is positioned directly on the detector(s) with light compression to limit patient motion. Patients receive an intravenous injection of the radiotracer (600–800 MBq ^99m^Tc-sestamibi for single-head MBI or 300 MBq for dual-head MBI-systems) in an antecubital vein contralateral to the breast lesion. Approximately 5–10 min after the injection of the radiotracer, standard planar images are performed for each breast in the craniocaudal (CC) and mediolateral oblique (MLO) projections. The acquisition time for each image is 8–10 min with a total acquisition time of approximately 40 min per study. If needed, additional images may be acquired (lateromedial or mediolateral view, anteroposterior view (axilla) or axillary craniocaudal view). These projections correspond to the standard projections used in MG (Fig. 1). For interpretation of the images a viewing system should be available which enables the adjustment of the image contrast and simultaneous display of the mammographic and scintigraphic images. The scintigraphic images are interpreted according to a functional BI-RADS classification, based on the guidelines of the Society of Nuclear Medicine (SNM) as shown in Table 2 and Fig. 2 [12]. Recently, a lexicon for the description of MBI images has been developed [22], based on familiar radiological BI-RADS lexicon terminology, as well as on the proposed BI-RADS-type lexicon for positron emission mammography (PEM).

**Table 2:** BI-RADS classification and MBI interpretation criteria according to SNM [12]

BI-RADS	MBI-interpretation
1-Negative	Homogeneous uptake
2-Benign	Patchy or diffusely increased uptake, often bilateral and correlating with MG anatomy
3-Probably benign	Multiple patchy areas of uptake, mild to moderate intensity
4-Suspicious for malignancy	Small focal areas of increased uptake
4a-Low	
4b-Intermediate	
4c-Moderate	
5-Highly suggestive of malignancy	Moderate to intense focal uptake with well-delineated contours

*BI-RADS* breast imaging-reporting and data system, *MBI* molecular breast imaging

## ^99m^Tc-sestamibi MBI-guided biopsy procedure

**Fig. 3:**
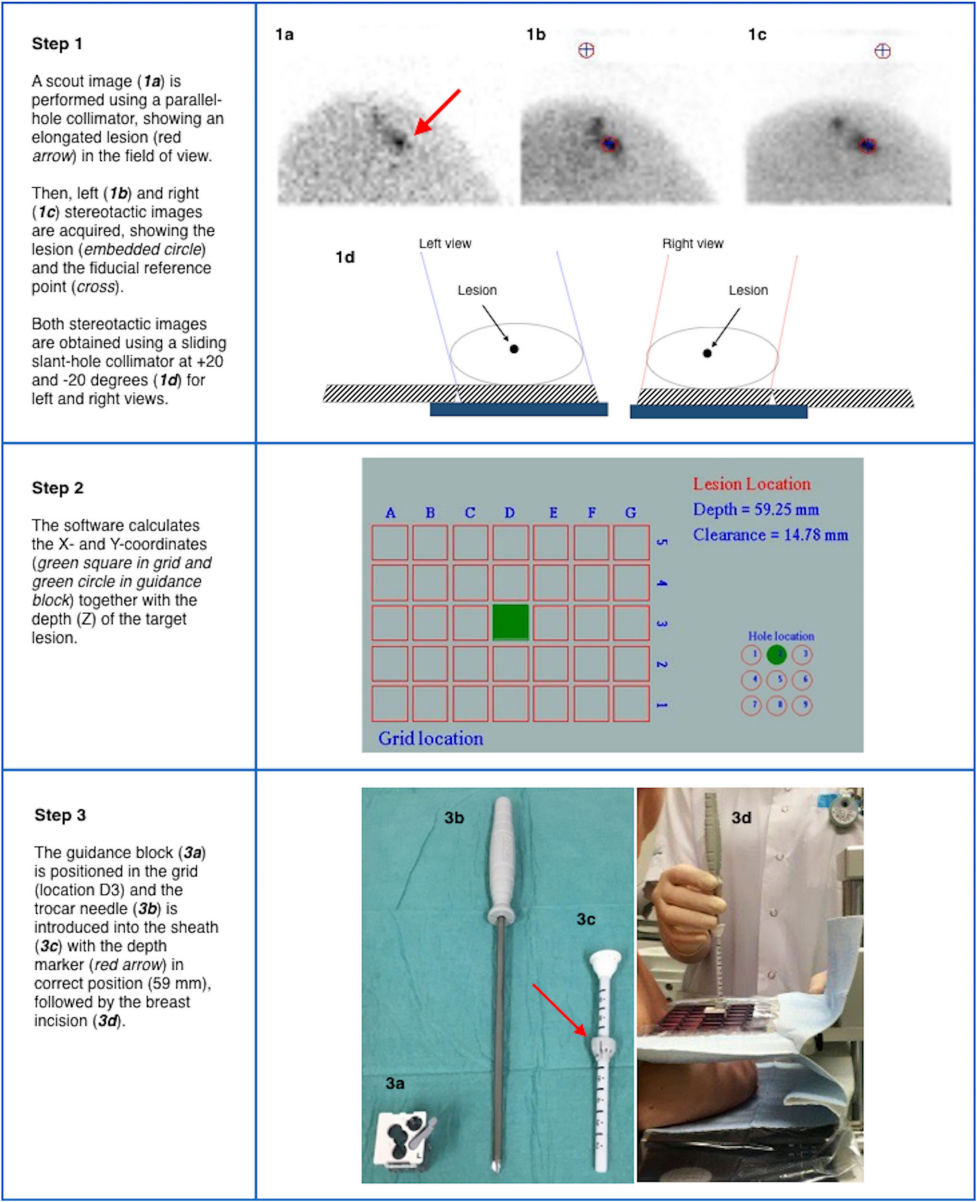

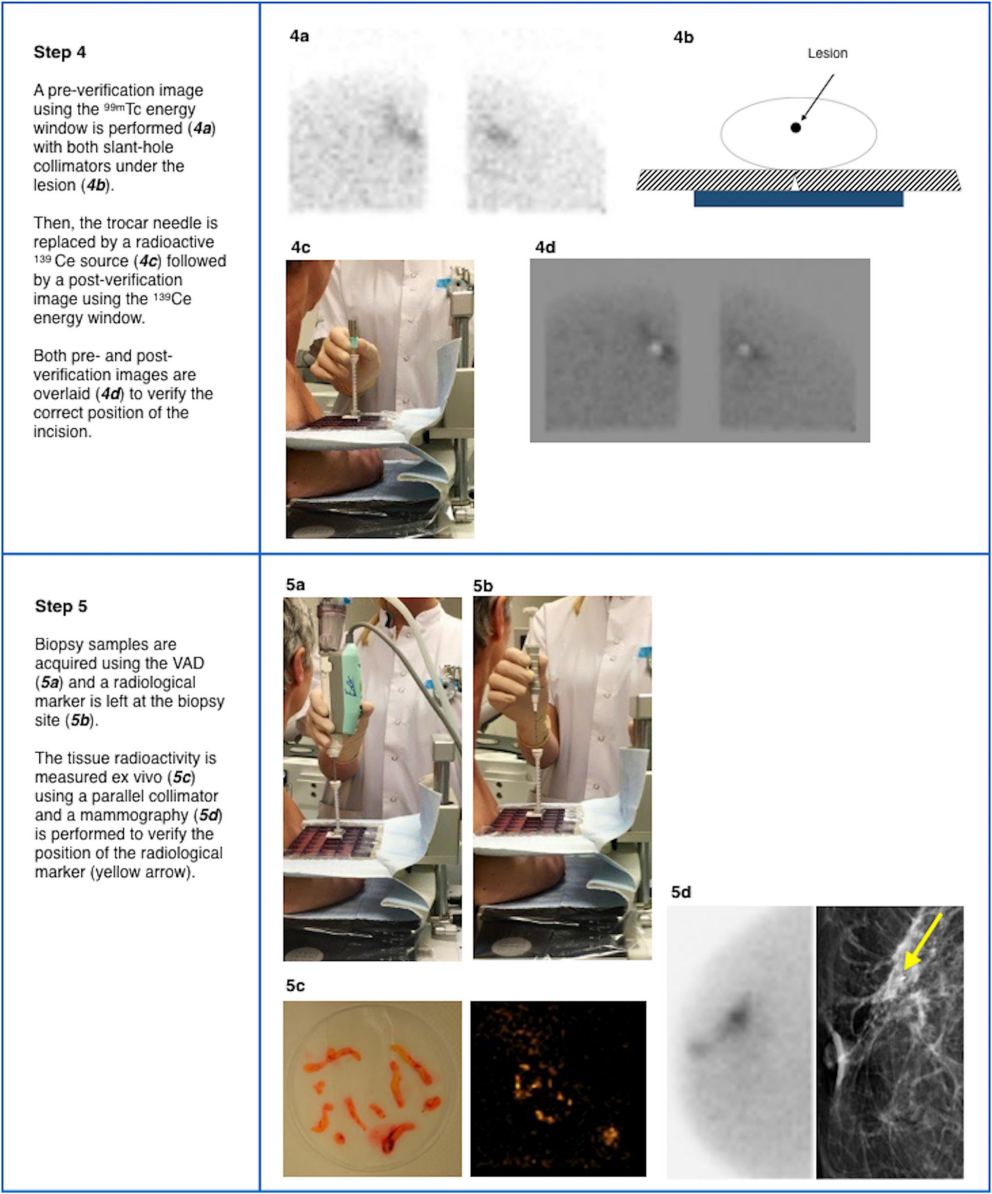
Procedure steps of ^99m^Tc-Sestamibi MBI-guided biopsy using a stereotactic localization system (GammaLoc^®^)

MBI-guided biopsy procedure is based on both preoperative imaging and intraoperative excision using ^99m^Tc-sestamibi as radiotracer for target tissue localization, according to the radioguided surgery concept [23]. To date, methodological aspects of PEM-guided biopsy using ^18^F-fluorodeoxyglucose (FDG) have been described [24], whereas no article has yet been reported the steps in MBI-guided breast biopsy using ^99m^Tc-sestamibi. For this latter modality, slant-hole collimator technology (GammaLōc^®^ MBI localization system, Dilon Technologies, Newport News, VA) is used to calculate the lesion depth using a single-head system [13]. Biopsy is performed with the patient in seated position. The breast is placed between the detector and the paddle (CorreLocator™, Dilon Technologies, US) with light compression to reduce patient motion. A fiducial source using Cerium-139 (^139^Ce) is imbedded into the compression paddle as spatial reference point for determining the position of the lesion. The patient is administered with approximately 600 MBq of ^99m^Tc-sestamibi into an arm vein contralateral to the breast lesion. Approximately 5 min after the injection, a scout image is performed using a parallel-hole collimator for positioning of the lesion. The breast lesion is in the exact position when it is assumed to be visible in the FOV of both the left and right stereotactic views. Subsequently, left and right stereotactic images are performed using a sliding slant-hole collimator (StereoView™, Dilon Technologies, US) for determining the grid localization (X, Y) and the depth (Z) of the lesion. Using this slant-hole collimator, 20 degree angle stereo views are required from both the left and right side (Fig. 3: Step 1). The location and the depth are clearly identified at the point where the angles intersect. Subsequently, the software (GammaLōc^®^, Dilon Technologies, US) calculates the X, Y, Z coordinates indicating the X and Y coordinates in the grid and the depth of the trocar needle in the guidance block (Fig. 3: Step 2). After injection of local anesthetic, the guidance block is placed in the paddle in the correct position. After the trocar needle is introduced into the sheath and the depth marker is set in the right position, the trocar needle is placed into the breast (Fig. 3: Step 3). Subsequently, a first image (pre-verification) is acquired in the energy window of ^99m^Tc with the needle in place. Afterwards, the trocar needle is removed and replaced by a radioactive ^139^Ce source followed by a second image (post-verification) using the energy window of ^139^Ce. Both pre- and post-verification images are acquired using both slant-hole collimators located under the lesion to verify the correct position of the needle (Fig. 3: Step 4). After this verification step, the actual biopsy is performed using a vacuum-assisted device (VAD). The VAD is composed of a large bore needle with an internal cutting trocar that rotates 360 degree around the axis of the needle cutting 6 specimens from the target lesion, which is vacuum aspirated into the sampling chamber. A radiological marker is left behind at the biopsy site to enable further lesion excision or follow-up. Tissue sample activity is measured ex vivo using the parallel-hole collimator, followed by histopathological analysis. Finally, MG is performed to verify the correct marker position (Fig. 3: Step 5).

## ^99m^Tc-sestamibi MBI-guided biopsy in comparison with MG, US and MRI-guided biopsy

**Fig. 4:**
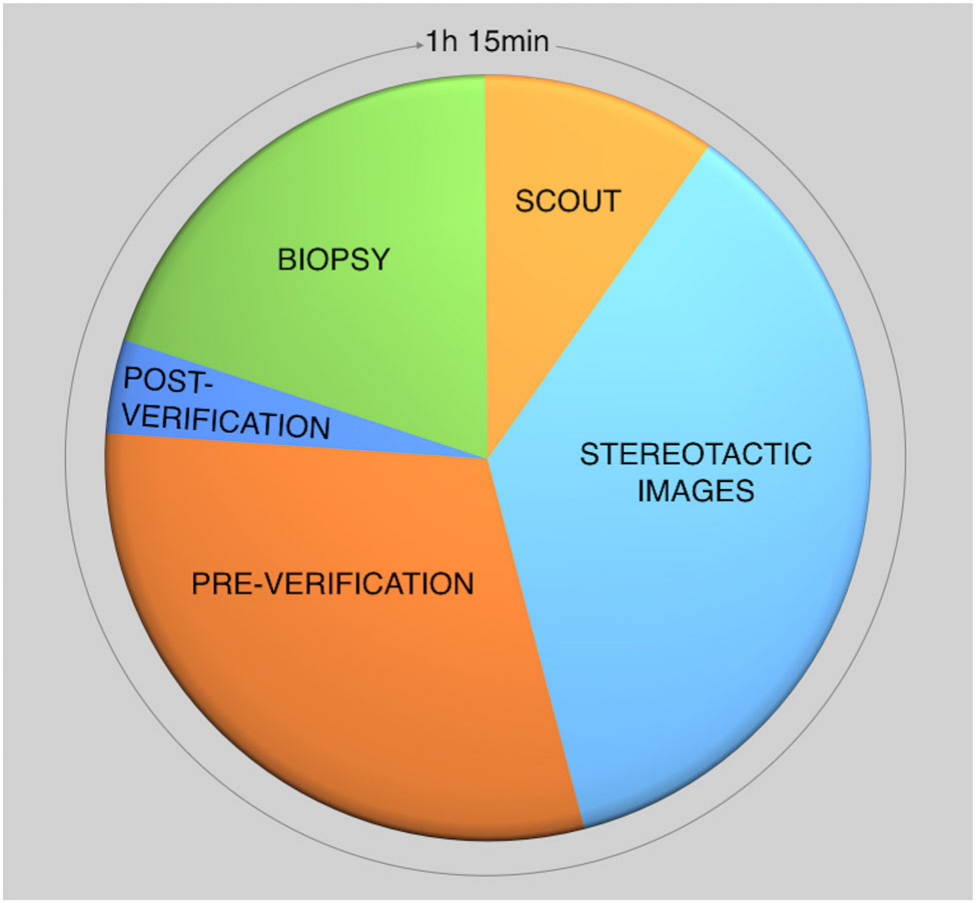
Procedure time of the different steps in ^99m^Tc-sestamibi MBI-guided biopsy

In recent years, percutaneous image-guided breast biopsy has gained importance as an alternative to surgical biopsy, mainly using sonographic, stereotactic, or MRI guidance. US-guided biopsy is the first technique of choice for sampling breast lesions. The sampling probe is placed behind the lesion to be biopsied and the verification of the correct needle placement is real-time. The main advantages of US-guided biopsy are its wide availability, lack of ionizing radiation and low costs [25]. Stereotactic biopsy is usually performed for sampling micro-calcifications and distortions not detected on US [26]. The patient is in upright or prone position and in both situations with compression of the breast [27, 28]. The prone position results in higher comfort for the patient, decreased likelihood of patient motion and less vasovagal reactions [29]. MRI-guided biopsy is principally performed when the breast lesion is occult both on US and MG [30]. The patient is in prone position with the breast located in a dedicated biopsy coil with compression in the mediolateral direction. The procedure time for MRI-guided biopsy is approximately 30–70 min [31, 32]. MRI-guided biopsy poses several challenges, such as the necessity to remove the patient from the magnet to perform the biopsy and the transient nature of the contrast enhancement. Furthermore, the access to the medial and posterior breast tissue is limited. An important limitation concerns the inability to verify the successful sampling of the target lesion, since tissue samples do not enhance ex vivo [33, 34]. As mentioned earlier, MBI is increasingly being used as adjunct modality to MG and US for detecting BC. In contrast to MG, MBI is a functional imaging technique that is not influenced by breast density and architectural distortion, regularly leading to the discovery of MG occult breast malignancies [18, 35]. For patients with occult or unclear breast lesions on MG/US but suspicious MBI, the possibility to use MBI-guided biopsy appears to be an excellent alternative to acquire representative tissue samples for histopathological analysis. To date, several MBI-guided biopsy methods have been described in the literature. In 2004, Coover et al. reported on a method to localise the lesion using a dedicated breast camera with an open biopsy paddle. The site of the lesion was identified using ^57^Co point source on the breast and the camera monitor in the persistent mode. Subsequently, two localization needles were placed into the site of the lesion followed by an open biopsy of the area where the two needles intersected. The authors reported a suspicious finding in 5 of 37 patients (13.7 %) with dense breasts and at high risk of breast cancer; biopsy revealed carcinoma in 3 out of 5 of these patients [36]. In 2006, Welch et al. reported on the development of a compact dedicated breast camera-guided stereotactic breast biopsy system. A fiducial marker containing 0.925 MBq of ^57^Co was mounted inside the top of the breast compression paddle as spatial reference point. An algorithm for determining the spatial location of the breast lesion was implemented in the software of the dedicated breast camera [37]. More recently, Weinmann et al. developed a conical slant hole (CSH) collimator for MBI-guided biopsy with dual-head CZT, improving the accuracy of lesion depth determination [38]. To our knowledge, no full studies have yet been reported on clinical validation of MBI-guided biopsy procedures. Based on our own clinical experience, the stereotactic biopsy method using the slant-hole collimator localization system as described here shows good patient acceptability. The procedure time is approximately 75 min, which is longer than the MG/US guided methods. The difference is mainly explained by the prolonged image acquisition which is necessary to accurately display lesion uptake of ^99m^Tc-sestamibi for subsequent stereotactic localization and biopsy (Fig. 4). However, procedure time is comparable to MRI guided biopsy. Complications are similar to those in other radiological biopsy methods such as syncope, hematoma formation and marker migration. Table 3 describes the clinical indications for MBI-guided biopsy. This biopsy method using ^99m^Tc-sestamibi is considered a complementary modality to MG/US-guided biopsy and an alternative to MRI-guided biopsy. It is principally indicated in patients with occult lesions on MG/US but suspicious on MBI (BI-RADS criteria 4–5) and occult after second-look US. Other possible indications include: (i) unclear lesions on MG/US but suspicious on MBI (BI-RADS criteria 4–5); (ii) failure of other biopsy methods. A potential future indication concerns optimization of primary tumour tissue sampling in patients with locally advanced breast cancer (LABC) by means of ^99m^Tc-sestamibi-guided targeted biopsy. In the literature non-correspondence between the core biopsy location and the area with highest metabolic activity in the tumour has been described for stage II/III breast cancer patients scheduled for neoadjuvant chemotherapy [39]. Since early and increased concentration of ^99m^Tc-sestamibi in breast carcinomas is associated with high proliferation rate, indicating more aggressive tumour behaviour [40], the sampling of the most proliferative parts of the tumour that correspond with highest uptake of ^99m^Tc-sestamibi in large heterogeneous tumours could result in more optimal therapy planning in patients with LABC. The advantages and disadvantages of the different biopsy methods are summarized in Table 4. The main advantage of MBI-guided biopsy compared to MRI-guided biopsy is the possibility to measure radioactivity of the tissue samples ex vivo, in this way verifying that the target lesion has been sampled successfully. However, biopsy may be difficult in lesions close to the pectoral muscle because they may not be completely visualized due to the vertical position of the patient in relation to the field of view of the camera.

**Table 3:** Indications for MBI-guided biopsy in clinical practice

Indication for MBI-guided biopsy
Occult lesions on MG/US but MBI-suspicious^a^ and occult after second look US
Unclear lesions on MG/US but MBI-suspicious^a^
Failure of earlier radiological biopsy
Future: targeted biopsy of large heterogeneous tumours in patients with locally advanced breast cancer

*MG* mammography, *US* ultrasound, *MBI* molecular breast imaging

^a^BI-RADS criteria 4 and 5

**Table 4:** Comparison of image-guided biopsy modalities

Biopsy method	Compression	Patient position	Advantages	Limitations
US-guided	No	Supine	Real time verification of needle position, fast, no ionizing radiation, low costs	Not useful for MC/distortions
Stereotactic	Yes	Upright/prone	Useful for MC/distortions, sample verification ex vivo possible (MC)	Ionizing radiation
MRI-guided	Yes	Prone	Useful for US and MG occult lesions, no ionizing radiation	High costs, long procedure time, limitation in claustrophobia, obesity and renal insufficiently, sample verification ex vivo not possible
MBI-guided	Yes (mild)	Upright	Useful for indeterminate/unclear lesions on MG/US, sample verification ex vivo possible	Lesions close to the pectoral muscle, ionizing radiation, long procedure time

*US* ultrasound, *MRI* magnetic resonance imaging, *MG* mammography, *MBI* molecular breast imaging, *MC* microcalcifications

In conclusion, MBI-guided biopsy represents an adjuvant tool to MG/US-guided biopsy and a promising alternative to MRI-guided biopsy. The principal application of this new biopsy method is in patients with occult or unclear lesions on MG and US that are suspicious on MBI (BI-RADS criteria 4 and 5). The future indication is in targeted biopsies in patients with large heterogeneous tumours. Further studies are needed to define the accuracy of this biopsy procedure.
